# Editorial: Mechanisms and therapeutic strategies in cellular injury and repair

**DOI:** 10.3389/fcell.2026.1789699

**Published:** 2026-02-17

**Authors:** Cheng Yuan, Jialiang Chen, Xiaorong Hu, Xiaolong Wang

**Affiliations:** 1 Department of Oncology, Yichang Central People’s Hospital and The First College of Clinical Medical Science, China Three Gorges University Yichang, Yichang, Hubei, China; 2 Tumor Prevention and Treatment Center of Three Gorges University and Cancer Research Institute of Three Gorges University Yichang, Yichang, Hubei, China; 3 Clinical Medical Research Center for Precision Diagnosis and Treatment of Lung Cancer and Management of Advanced Cancer Pain of Hubei Province, Yichang, Hubei, China; 4 Central Laboratory, The First College of Clinical Medical Science, China Three Gorges University and Yichang Central People’s Hospital, Yichang, Hubei, China; 5 Department of Urology, Shanghai Ninth People’s Hospital, Shanghai Jiaotong University School of Medicine, Shanghai, China; 6 Department of Cardiology, Zhongnan Hospital of Wuhan University, Wuhan, China; 7 Institute of Myocardial Injury and Repair, Wuhan University, Wuhan, China; 8 Lewis Katz School of Medicine, Temple University, Philadelphia, United States

**Keywords:** cellular injury, ferroptosis, mechanisms, repair, therapeutic strategies

Cellular injury is a common response to heterogeneous insults, such as ischemia-reperfusion, toxicants, metabolic byproducts, infection, and oncologic stress. However, this response often converges on a limited set of interconnected hubs: oxidative stress, mitochondrial dysfunction, disrupted Ca^2+^ homeostasis, immune activation, and regulated cell death. The nine articles collected in this Research Topic cover topics ranging from heart and kidney function to cancer, reproductive toxicology, and physiological remodeling. Together, they illustrate how advances in mechanisms, biomarkers, and intervention design can be applied to shared molecular logic while still respecting tissue-specific contexts.

A central theme across this Research Topic is the role of the oxidative stress-lipid peroxidation axis in driving ferroptotic vulnerability in renal epithelial injury. In an infection context, Lu et al. report that *Pseudomonas aeruginosa* promotes lipid peroxidation via a secreted 15-lipoxygenase and compromises epithelial antioxidant defense by reducing GPX4 through lysosomal chaperone-mediated autophagy, thereby triggering ferroptosis. Strikingly, macrophages counter this vulnerability: M1 macrophage-derived NO, generated by iNOS, remotely suppresses epithelial ferroptosis even when thiol-based antioxidant capacity is limited. Complementing the infection-driven pathway, Zhang et al. link the reactive dicarbonyl methylglyoxal (MG) to kidney injury, showing that MG increases ROS, depletes glutathione, elevates lipid peroxidation, and triggers ferroptosis in renal tubular epithelial cells. Together, these studies expand the range of upstream routes into ferroptosis, from microbial effectors to metabolic carbonyl stress, and highlight translational opportunities, including limiting lipid peroxidation, preserving GPX4 activity, and modulating immune-derived protective signals.

Ischemia-reperfusion injury (IRI) adds another dimension by coupling metabolic stress to immune-mediated amplification. Through integrated bulk and single-cell transcriptomic analyses across multiple IRI organ datasets, Zheng et al. identify ANXA1 and ARG2 as early and consistently upregulated genes linked to distinct T-cell infiltration signatures. Beyond proposing ANXA1 and ARG2 as pan-organ biomarkers of IRI, the study uses drug prediction and molecular dynamics to nominate candidate agents (hydrocortamate and NS6180) that may target T-cell proliferation programs and validates key gene expression changes in a renal IRI model. This “multi-model, multi-omic, druggable-target” workflow is a useful blueprint for moving from injury-associated signatures to testable intervention hypotheses.

Two mechanistically aligned contributions focus on myocardial I/R injury and emphasize mitochondria-centered control of the redox state and regulated cell death. Chen et al. show that gypensapogenin I reduces infarct size and improves cardiac function while suppressing oxidative stress, ferroptosis, and PANoptosis. In a complementary study, Chen et al. report that *Morinda officinalis* oligosaccharides attenuate oxidative stress and mitochondria-associated ferroptosis, with evidence implicating inhibition of NOX4 and enhancement of mitochondrial antioxidant capacity, including promotion of mitoGPX4. Together, these studies highlight NOX-mitochondria circuits (NOX2/AMPK and NOX4/mitoGPX4) as convergent leverage points where redox, mitochondrial quality control, and multiple death programs intersect.

This Research Topic also extends mitochondria-directed protection into reproductive toxicology. Varias et al. demonstrate that melatonin and agomelatine counter ivermectin-induced spermatogonial apoptosis by suppressing oxidative stress and Ca^2+^ overload while restoring mitochondrial membrane potential, mitochondrial mass, and oxidative phosphorylation. By placing redox and Ca^2+^ dysregulation upstream of mitochondrial failure and cell loss, the study reinforces a broader therapeutic principle reflected across this Research Topic: interventions that stabilize mitochondrial function can shift injury trajectories toward recovery.

A second major focus of this Research Topic is precision phenotyping to capture cellular heterogeneity and dynamic remodeling, thereby enabling earlier and more individualized interventions. Rong and Zhou review how single-cell and spatial multi-omics are transforming the study of cardiac metabolic reprogramming, revealing cell-type-specific metabolic states, intercellular communication, and immunometabolic coupling that are masked in bulk measurements. In oncology, Zhao et al. demonstrate that multiparametric MRI can provide noninvasive biomarkers linked to cellular injury and proliferation in nasopharyngeal carcinoma; diffusion and perfusion/permeability metrics predict short-term treatment response and correlate with Ki-67. Together, these contributions emphasize that mechanistic targeting and clinical translation are strengthened when quantitative readouts can stratify injury states and monitor early response.

Finally, repair encompasses physiological remodeling programs and post-injury healing. Peng et al. map miRNA dynamics across lactation and involution stages in the mammary glands of dairy goats and validate a miR-423-3p/IGF1R axis that restrains epithelial proliferation, induces cell-cycle arrest, and promotes apoptosis via PI3K/Akt suppression. Beyond its system-specific insights, the study provides a tractable regulatory module that links transcriptomic remodeling to functional outcomes, an approach that can be extended to other tissues where controlled proliferation and apoptosis determine the quality of repair.

Collectively, the studies in this Research Topic highlight priorities for next-stage progress: i. treating regulated cell death as an interacting network in which ferroptosis, PANoptosis, apoptosis, and immune-mediated damage are shaped by the mitochondrial redox state and intercellular signaling; ii. integrating causality with measurement by pairing genetic or pharmacological perturbations with single-cell, spatial, quantitative imaging, and functional readouts; and iii. accelerating translation through biomarker-guided stratification and rational combination strategies that target convergent hubs while respecting tissue-specific context.

